# Hazelnut Shell Biorefinery for Bioactive CMC Films: Sequential Polyphenol and Cellulose Recovery and Wax-Modulating Performance

**DOI:** 10.3390/foods15122166

**Published:** 2026-06-16

**Authors:** Sarmad Ahmad Qamar, Simona Piccolella, Luana Izzo, Emilio Di Stasio, Giampaolo Raimondi, Severina Pacifico

**Affiliations:** 1 Department of Environmental, Biological & Pharmaceutical Sciences and Technologies, University of Campania ‘Luigi Vanvitelli’, Via Vivaldi 43, 81100 Caserta, Italy; sarmadahmad.qamar@unicampania.it (S.A.Q.); simona.piccolella@unicampania.it (S.P.); 2 Department of Pharmacy, University of Naples ‘Federico II’, Via Domenico Montesano 49, 80131 Naples, Italy; luana.izzo@unina.it; 3 Department of Agriculture, University of Naples ‘Federico II’, Via Università 100, 80055 Naples, Italy; emiliodistasio@gmail.com (E.D.S.); giampaolo.raimondi@unina.it (G.R.)

**Keywords:** hazelnut shell, polyphenolic compounds, carboxymethyl cellulose (CMC), bioactive packaging, agro-industrial waste valorization

## Abstract

The valorization of lignocellulosic residues into bioactive and biodegradable materials offers a sustainable route for functional food packaging. In this study, hazelnut shells were exploited through an integrated process enabling the integrated recovery of polyphenols and cellulose. Polyphenols were extracted via hot water, liquid–liquid partitioning, and column chromatography, yielding a purified bioactive fraction. The residual biomass after polyphenol recovery was used for cellulose extraction (approximately 23% *w*/*w*) and converted into carboxymethyl cellulose (CMC) with a degree of substitution (DS) of 0.77. Active CMC films incorporating polyphenolic extracts exhibited improved mechanical performance, reaching tensile strengths of about 78 MPa and elongation at break values above 20%, while reducing water solubility to approximately 31%. The addition of carnauba wax further enhanced water resistance while modulating flexibility and stiffness. Attenuated Total Reflectance-Fourier Transform Infrared spectroscopy (ATR-FTIR) and scanning electron microscopy (SEM) analyses confirmed the conversion of crystalline cellulose into amorphous CMC and the successful incorporation of additives within the polymer matrix. The resulting films showed tunable mechanical, optical, and barrier properties, along with UV-blocking and antioxidant activity. These findings demonstrate that hazelnut shell-derived CMC films enriched with polyphenols and carnauba wax represent promising candidates for a sustainable platform for active food packaging applications, supporting a circular waste-to-value approach.

## 1. Introduction

The environmental impact associated with conventional plastics has become a critical global concern, driving the scientific community and industry toward the development of biodegradable and renewable alternatives. Petroleum-based polymers, although highly effective in ensuring food protection and extending shelf-life, accumulate in terrestrial and marine ecosystems due to their limited degradability, contributing significantly to long-term pollution and resource depletion. This scenario has intensified interest in converting agri-food residues into biobased materials, a strategy that aligns with circular economy principles and offers a sustainable route to reduce plastic waste generation while valorizing underexploited biomass streams [1,2].

Food supply chain byproducts represent an abundant and renewable source of lignocellulosic materials and bioactive compounds. Among natural biopolymers, cellulose is particularly attractive due to its renewability, mechanical strength, film-forming ability, and broad chemical modifiability [3,4,5,6]. However, the high crystallinity of native cellulose limits its solubility and processability, necessitating chemical derivatization to obtain more versatile materials. Carboxymethylcellulose (CMC), a water-soluble cellulose derivative, has gained increasing relevance in food packaging applications owing to its biodegradability, biocompatibility, and tunable physicochemical properties [7]. CMC-based films are transparent, flexible, and easy to process, yet their intrinsic hydrophilicity restricts their barrier performance, particularly under humid conditions, which remains a major limitation for practical applications.

In parallel, the development of active packaging materials has emerged as a promising strategy to enhance food quality, safety, and shelf-life through the incorporation of natural functional additives. Polyphenols are especially appealing due to their strong antioxidant, antimicrobial, and UV-blocking activities [8,9,10]. Their integration into biodegradable polymer matrices can impart multifunctionality, enabling films to act not only as passive barriers but also as active protectants against oxidative degradation and light-induced deterioration. However, challenges persist regarding the efficient recovery of polyphenols from biomass, their stability during processing, and their homogeneous dispersion within hydrophilic matrices such as CMC without compromising mechanical integrity [9,10].

Within this broader context, nut-processing industries represent a particularly relevant source of bioactive-rich lignocellulosic residues. Italy, and especially the Campania region, is one of Europe’s leading producers of hazelnuts (*Corylus avellana* L.), generating substantial quantities of shells that remain largely underutilized despite their high content of cellulose and phenolic compounds [11]. The strong regional concentration of hazelnut cultivation, combined with the increasing interest in sustainable valorization pathways, makes this biomass a worthy candidate for integrated biorefinery approaches aimed at producing functional packaging materials. Hazelnut shells, in particular, constitute an abundant and underexploited byproduct of the nut processing chain and offer a unique combination of cellulose and polyphenolic compounds suitable for dual valorization. Efficiently exploiting both recovered material streams within a single processing framework offers a dual advantage: reducing agro-industrial waste and generating high-value functional biomaterials. In fact, although previous studies have reported the extraction of either polyphenols or cellulose from hazelnut shells and related agricultural residues [12,13,14], to the best of our knowledge, no study to date has implemented a fully integrated approach that recovers both fractions from the same hazelnut biomass and employs them in the fabrication of active packaging films. Moreover, only limited research has explored the use of natural hydrophobic agents such as carnauba wax to overcome the moisture sensitivity of CMC-based films and enhance their barrier properties [4].

The present study addresses these gaps by developing bioactive films derived from hazelnut shell biomass through a comprehensive “waste-to-value” strategy. The novelty of the approach lies in the integrated valorization of the same agro-industrial residue as a source of both cellulose, being converted into CMC as the structural film-forming matrix, and polyphenols, used as native bioactive additives to impart antioxidant and UV-blocking functionality. Carnauba wax was introduced as a natural hydrophobic component to improve water resistance and modulate mechanical behavior. The resulting films were thoroughly characterized in terms of structural, morphological, physical, mechanical, and barrier properties, and their biofunctional performance was evaluated through antiradical assays (ABTS and DPPH) and UV-blocking analysis. This work demonstrates the potential of hazelnut shell-derived CMC composites as sustainable, high-performance materials for active food packaging applications and highlights the value of integrated biorefinery approaches for the valorization of agro-industrial residues.

## 2. Materials and Methods

### 2.1. Chemicals and Reagents

All the solvents used were purchased from VWR International (Milan, Italy), except for ultrapure water, obtained from a Milli-Q system (Millipore, Bedford, MA, USA). The chemicals and reagents used, as well as Amberlite XAD-4 and the analytical standard used in mass spectrometry investigation, were purchased from Merck Life Science S.r.l. (Milan, Italy).

### 2.2. Recovery and Characterization of Polyphenolics from Hazelnut Shells

**Figure 1:**
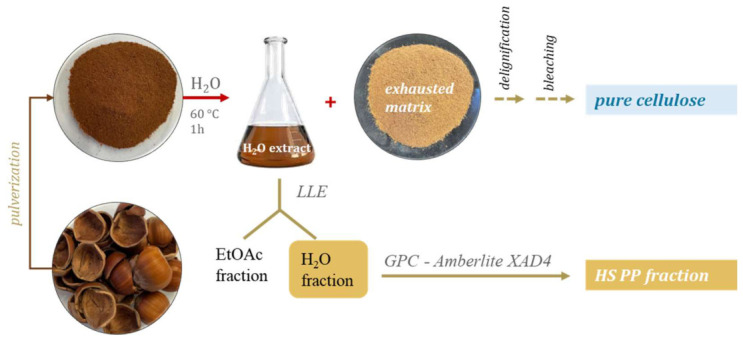
Schematic representation of the fractionation of hazelnut shells (HSs). Ground hazelnut shells were first subjected to aqueous extraction to recover water-soluble compounds. The crude extract was purified via liquid–liquid extraction (LLE) with ethyl acetate (EtOAc). Gel permeation chromatography (GPC) on Amberlite XAD-4 resin was utilized for recovering the polyphenolic fraction (PF). The remaining solid residue was processed for cellulose isolation.

Hazelnut shells were collected from a local hazelnut processing facility in Avellino, Italy, and used as received for subsequent extraction (Figure 1), after visual inspection to remove any evident foreign material or contaminated particles. The shells were stored in paper bags away from light and heat sources until use. Before extraction, the shells were coarsely ground to increase surface area and facilitate solvent penetration. Then, an aliquot of hazelnut shells (50 g) underwent aqueous extraction (500 mL; solid-to-solvent ratio 1:10, g:mL) at 60 °C under continuous magnetic stirring for 1 h. The solvent was removed under vacuum by a rotary evaporator (Heidolph Hei-VAP Advantage, Schwabach, Germany), yielding 2.6 g of crude extract, corresponding to a 5.2% recovery relative to the initial material. The latter was redissolved in water and subjected to liquid–liquid extraction (LLE) with ethyl acetate (2:1 ratio, repeated three times) with vigorous mixing and subsequent phase separation. The ethyl acetate fraction (0.248 g, 9.5% of the aqueous extract) was not analyzed further in this study, as it was enriched in lipid components. The residual aqueous phase was further purified using Amberlite XAD-4 resin. The resin was equilibrated with water (resin-to-solution ratio 1:10, *w*:*v*) to remove polar impurities, followed by elution of the target fraction with methanol (resin-to-solvent ratio 1:5, *w*:*v*). The alcoholic fraction was concentrated under reduced pressure, yielding 220 mg of purified material, corresponding to 8.5% of the initial aqueous extract. The alcoholic fraction was subsequently characterized using spectroscopic and high-resolution mass spectrometric techniques to elucidate its chemical composition and was then employed for the fabrication of bioactive films. The isolation of cellulose from the residual hazelnut shell biomass after polyphenol extraction is described in Section 2.3.

#### 2.2.1. Spectroscopic Characterization of the Polyphenol Fraction

The polyphenol fraction obtained from hazelnut shells was characterized using UV-Vis and ATR-FTIR spectroscopic techniques. UV-Vis absorption spectra were recorded in the range of 200–800 nm by a Cary 100 spectrophotometer (Agilent Technologies, Milan, Italy) against a blank. Measurements were performed in three independent replicates. ATR-FTIR spectra were acquired in the 4000–500 cm^−1^ range using an IRXross FTIR spectrophotometer (Shimadzu, Tokyo, Japan), with a spectral resolution of 4 cm^−1^ and 45 scans per sample. To this aim, the dried extract was directly placed on the ATR-FTIR crystal and analyzed without further sample preparation. Spectral data were processed using the LabSolutions IR software (v.1.60, Shimadzu, Tokyo, Japan).

#### 2.2.2. UHPLC-Q-Orbitrap HRMS Analysis

Polyphenolic fraction was analyzed using a Vanquish DAD-Flex UHPLC system (Thermo Fisher Scientific, Waltham, MA, USA) equipped with a refrigerated autosampler (WPS-3000), micro-degasser (GPL-3400RS), thermostatic column oven (TCC-3100), and binary pump (HPG-3400RS). Chromatographic separation was performed on a Kinetex Biphenyl column (100 × 2.1 mm, 2.6 µm) maintained at 30 °C (Phenomenex, Castel Maggiore, Italy). The mobile phases included (A) 0.1% formic acid (HCOOH) in water, and (B) 0.1% HCOOH in methanol. A gradient program was applied at 0.4 mL/min over 13 min, starting at 100% A (0–0.5 min), decreasing to 30% A at 1.5 min and to 15% A at 8 min, and then returning to initial conditions (100% A) at 11 min, which were maintained until 13 min. The injection volume was 5 µL. Mass spectrometry was carried out on a Q-Exactive Orbitrap (Thermo Fisher Scientific) in negative electrospray ionization (ESI-) mode over an *m*/*z* range of 80–1200. Data were acquired in both full MS and all-ion fragmentation (AIF) modes. The resolving power was 70,000 FWHM with an AGC target of 1 × 10^6^ for full MS, and 17,500 FWHM with an AGC target of 1 × 10^5^ for AIF. The maximum injection time was 200 ms. Collision energies of 15, 30, and 45 eV were applied. Data processing was performed using Quan/Qual Browser Xcalibur software (v. 3.1.66.10, Thermo Fisher Scientific). For targeted analysis, polyphenolic compounds were identified and quantified by comparison with analytical standards injected at concentrations ranging from 5 ppm to 0.0024 ppm, prepared by 1:2 serial dilutions in 0.1% HCOOH in methanol. Untargeted identification was based on accurate mass measurements and comparison with theoretical masses.

### 2.3. Recovery of Cellulose from Leftover Hazelnut Shell Biomass

A separate batch of dried and ground hazelnut shell biomass (100 g) was first subjected to the aqueous extraction procedure described in Section 2.2 to remove the polyphenolic fraction. The resulting residual solid biomass was then used for cellulose extraction following a pre-established method [4,5,6]. Briefly, the residual biomass was treated with a sodium hydroxide solution (5%, *w*/*v*; 1 L) at 80 °C for 4 h in a thermostated water bath to promote lignin removal. After alkaline treatment and neutral washing, delignification was carried out using an acidic sodium chlorite solution (1.4%, *w*/*v*; 1 L). The pH was adjusted to 3.5–4.0 with glacial acetic acid before heating, and the suspension was maintained at 75 °C for 3 h under controlled conditions. During the reaction, the pH was monitored and maintained below 4.0. The solid fraction was then recovered and extensively rinsed with distilled water. This oxidative treatment was repeated twice to ensure efficient lignin removal and improve cellulose purity. The yield of cellulose was 22.7% (*w*/*w*) relative to the dry weight of hazelnut shell feedstock.

### 2.4. Functionalization of Hazelnut Shell Cellulose to Carboxymethylcellulose

The functionalization of hazelnut shell cellulose (HS-C) into carboxymethylcellulose (HS-CMC) was carried out through a two-step procedure consisting of alkalization followed by etherification, according to Haleem et al. [15], with minor modifications. Briefly, 5 g of cellulose was dispersed in 150 mL of isopropanol, and alkalization was achieved by the dropwise addition of 20 mL of sodium hydroxide solution (50%, *w*/*v*) under continuous stirring at room temperature. Subsequently, 10 g of monochloroacetic acid was introduced into the reaction system to initiate carboxymethylation. The reaction mixture was maintained at 50 °C for 4 h under magnetic stirring. Upon completion, the solid product was separated by filtration and suspended in 100 mL of ethanol to terminate the reaction. Neutralization was performed using diluted acetic acid. The resulting CMC was extensively washed with 70% ethanol to remove residual reagents and by-products, then freeze-dried and weighed. The percentage yield was calculated as the ratio of the dry CMC mass to the initial cellulose mass used in the reaction. The DS was determined by titration according to Qamar et al. [4]. Briefly, the sample was suspended in 95% ethanol, followed by the addition of 5 mL of 2 M hydrochloric acid solution. After stirring for 10 min at room temperature, the mixture was heated to boiling and allowed to cool, and the solid fraction was separated by filtration. The residue was thoroughly washed with ethanol and methanol to remove excess acid and impurities, and then oven-dried. For titration, 0.5 g of dried CMC was dissolved in 100 mL of distilled water under continuous stirring and treated with 25 mL of 0.5 M sodium hydroxide solution. The mixture was boiled for 20 min and subsequently titrated with 0.3 M HCl solution using phenolphthalein as an indicator. The endpoint was identified by the disappearance of the pink coloration. The DS was calculated from the amount of acid consumed per gram of sample, taking into account the molecular weight of the anhydrous glucose unit (162 Da) and the mass increase associated with each carboxymethyl substitution (58 Da).

### 2.5. Development of Bioplastics from Hazelnut Shell CMC

The bioplastic films were prepared by mixing 2.80 g/100 mL of HS-CMC in distilled water at ambient temperature (~25 °C) using a hot-plate magnetic stirrer to get a homogenous suspension. Sorbitol (1.20 g/100 mL) was added to the film-forming solution as a plasticizer, and the mixture was heated at 50 °C for 30 min under continuous stirring. The solution was added with hazelnut shell polyphenols (40 and 80 mg/100 mL) to prepare active films. In addition, a control film was prepared without the addition of polyphenolic extract. To further improve the hydrophobic properties of the films, two separate prototypes were prepared by incorporating carnauba wax inside the film-forming solution. For this purpose, a wax-containing emulsion was prepared by dissolving agar (0.5 g/100 mL) in water at ~85 °C until a clear transparent solution was obtained. Then, 1 mL of Polysorbate 40 (Tween 40) was added as an emulsifier, followed by the gradual incorporation of 1 g of melted carnauba wax (CW). The resulting mixture was sonicated for 30 min to get a homogeneous emulsion. Subsequently, two separate film-forming solutions containing hazelnut shell polyphenols (40 mg/100 mL and 80 mg/100 mL) were prepared and supplemented with the wax emulsion, maintaining a final wax concentration of 400 mg/100 mL. All film-forming solutions were cast into flat plates and dried at 40 °C for 24 h. Then, they were gently scratched using a spatula to proceed with further investigations. The films were designated as F0 (control—without hazelnut shell polyphenol extract), F1 (40 mg/100 mL extract), F2 (80 mg/100 mL extract), F3 (40 mg/100 mL extract + 400 mg/100 mL CW), and F4 (80 mg/100 mL extract + 400 mg/100 mL CW) (Table 1).

### 2.6. Characterization

#### 2.6.1. Structural, Morphological, and Thermal Characterization

The hazelnut shell cellulose, resulting CMC, and produced bioplastics films were characterized using Attenuated Total Reflectance-Fourier transform infrared (ATR-FTIR) spectroscopy (IRXross Shimadzu, Tokyo, Japan) to evaluate major functional groups. The clean and dried samples were placed directly under the infrared beam, and spectra (4000–400 cm^−1^) were obtained in absorbance mode using 45 scans with a resolution of 0.25 cm^−1^. The total crystallinity index (TCI), the lateral order index (LOI), and the hydrogen bond intensity (HBI) were calculated using the absorbance ratios between the characteristic bands associated with C-H bending and C-H stretching, CH_2_ bending and β-glycosidic bond vibration, and O-H stretching and C-H bending vibrations, as previously reported [6]. X-ray diffraction patterns were recorded using a GNR instrument (Novara, Italy) equipped with a Theta/Theta geometry and operating with Cu Kα radiation (λ = 1.5406 Å). The scans covered a 2θ interval from 2° to 40°, with a step of 0.02°/min.

The morphologies of hazelnut shell cellulose, resulting CMC, and newly produced bioplastics, both surface and cross-sectional morphologies, were recorded using an ultra-high-vacuum model of SEM (Quanta 200 FEG-152 FEI, Eindhoven, The Netherlands), and high-definition (HD) images were taken at an accelerating voltage of 20 kV.

Thermal behaviour was investigated by thermogravimetric analysis. Approximately 20 mg of sample was heated from 30 °C up to 600 °C at a constant rate of 10 °C/min under a nitrogen flow of 20 mL/min using a TGA 4000 system (PerkinElmer, Milan, Italy).

#### 2.6.2. Physical Properties of Films

Various physical properties of the films, including thickness, color (whiteness and yellowness indices), transparency, moisture content, solubility, water vapor transmission rate (*WVTR*), and water vapor permeability (*WVP*), were evaluated. Film thickness was determined using a digital micrometer (0–25 mm/0–1 and 0.001 mm/0.0001 resolution). Color measurements were performed with a Chroma Meter CR-5 (Konica Minolta, Tokyo, Japan), recording L* (*lightness*), a* (*red-green*), and b* (*yellow-blue*) values. These parameters were then used to calculate the yellowness index (*YI* = 142.86 b* L^−1^) and whiteness index (*WI* = 100 − [(100 − *L*)^2^ + *a*^2^ + *b*^2^]^0.5^) according to Łopusiewicz et al. [16]. Transparency was assessed by measuring the transmittance at 600 nm (*T*_600_) using a UV-Vis spectrophotometer (Cary 100, Agilent Technologies, Milan, Italy). Film specimens of appropriate size were mounted in the sample holder, and three measurements were recorded for each sample. The transparency index was calculated as *TI* = log*T*_600_/thickness, with results reported as mean ± SD. Moisture content was determined by weighing film samples (2 × 2 cm^2^, *W*_1_) and drying them in a hot-air oven until a constant weight (W_2_) was reached. The percentage moisture content was calculated as [(*W*_1_ − *W*_2_)/*W*_1_] × 100. Solubility was assessed by soaking the dried films in 20 mL of distilled water overnight. Insoluble residues were collected on pre-weighed filter paper and dried to constant weight (*W*_3_), and solubility is expressed as [(*W*_2_ − *W*_3_/*W*_2_] × 100 [17]. *WVTR* and *WVP* were determined following the method of Qamar et al. [4]. Briefly, impermeable glass cups, containing silica desiccant, were sealed with the films and placed in a desiccator alongside a cup of distilled water to establish a controlled relative humidity environment (approximately 0% inside the cups, 50% in the desiccator, at 25 °C). Cups were weighed after 24 and 48 h to calculate *WVTR* [*WVTR* = Δ*W*/(*A* × *t*)], where *ΔW* is the weight change (g), A is the film-covered area (m^2^), and t is the exposure time (h). Water vapor permeability was then calculated as *WVP* = (*WVTR* × *L*)/Δ*P*, where *L* is the film thickness and Δ*P* is the vapor pressure difference across the film.

#### 2.6.3. Mechanical Properties of Films

Mechanical parameters: tensile strength, elongation at break, and Young’s modulus were calculated using UTM (EZ-SX Shimadzu, Kyoto, Japan). The samples were prepared by cutting films into dog-bone shapes [length 5 cm, width 2 cm (wider ends), and 1 cm (middle)]. Film thickness was measured using a digital micrometer with a measuring range of 0–25 mm and a resolution of 0.001 mm. No specific preconditioning step was applied before mechanical testing. The machine was calibrated prior to analysis using the manufacturer-provided instructions to ensure the accuracy and reliability of the results. The samples were carefully attached to holding grips, ensuring proper alignment to minimize chances of bending/twisting during analysis. The machine was operated at a crosshead speed of 5 mm/min using a 100 N load cell. The measurements were obtained in triplicate to calculate mechanical parameters, tensile strength (*TS = Force/area*), Young’s modulus (*YM = stress/strain*), and elongation at break (*EB% = change in length/original length* × 100), as previously detailed [4].

#### 2.6.4. UV-Blocking Properties of Films

The films were analyzed for their UV-blocking potential by measuring optical transmittance from 200–800 nm using a UV-Vis spectrophotometer (Cary 100, Agilent-Tech, Milano, Italy). Before analysis, films were cut into appropriate dimensions (3.0 × 0.5 cm, l × w) to place in the sample holder. Transmittance percentage was recorded across the UV and visible regions to determine their optical clarity and potential UV-blocking characteristics [5]. All measurements were recorded in triplicate to ensure reproducibility.

#### 2.6.5. Antioxidant Properties of Films

The antioxidant activity of the newly developed films was evaluated using ABTS [2,2′-azino-bis(3-ethylbenzothiazoline-6-sulfonic acid)] and DPPH (2,2-diphenyl-1-picrylhydrazyl) radical scavenging assays [18]. For each film, three samples were cut into discs of 5 mm diameter and placed into individual wells of a 96-well microplate. A volume of 300 µL of radical solution (ABTS or DPPH) was added to each well, while wells containing only the radical solution served as blanks. Trolox solutions (2, 4, 8, 16, and 32 µM) were included as positive controls. The plates were incubated for 90 min, and absorbance was measured every 15 min to track the kinetics of radical scavenging. Absorbance readings were taken at 734 nm for ABTS and 515 nm for DPPH using a spectrophotometer (Mobi µ2, MicroDigital Co., Ltd., Seongnam, Republic of Korea). Radical scavenging activity was calculated as the percentage inhibition, based on the reduction in absorbance relative to the blank, as previously reported [5].

### 2.7. Statistical Analysis

Data are expressed as mean ± standard deviation (SD) from three independent experiments. Differences between groups were analyzed using one-way ANOVA, followed by Tukey’s post hoc test for multiple comparisons. A *p*-value < 0.05 was considered statistically significant.

## 3. Results and Discussion

### 3.1. Chemical Characterization of Hazelnut Shell Polyphenolic Extract

**Figure 2:**
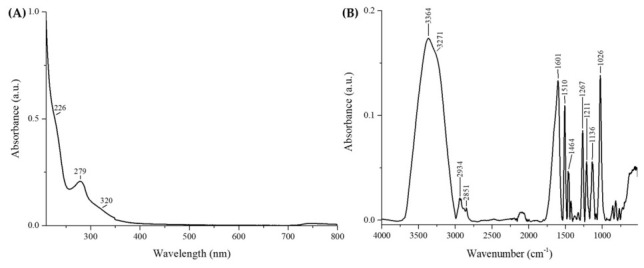
(**A**) UV-Vis and (**B**) ATR-FTIR spectra of the obtained hazelnut shell extract.

The hazelnut shell biomass was initially subjected to hot water extraction to recover secondary metabolites, yielding a crude aqueous extract, rich in soluble phenolic compounds. This extract was further purified through a two-step procedure to isolate the bioactive polyphenolic fraction. First, a liquid–liquid extraction (LLE) with ethyl acetate was performed to remove lipidic and highly polar components. The resulting aqueous phase was subsequently fractionated by GPC, using Amberlite XAD-4 resin, to eliminate low-molecular-weight sugars, amino acids, and other hydrophilic impurities. The adsorbed fraction was finally eluted with methanol, affording an enriched polyphenolic extract. Spectroscopic (UV-Vis and ATR-FTIR; Figure 2) and high-resolution mass spectrometric (UHPLC-Orbitrap HR-MS) analyses confirmed that this purified fraction was dominated by phenolic acids, flavonoids, and related polyphenolic derivatives.

The UV-Vis spectrum exhibited characteristic absorption bands at 226 nm, 279 nm, and 320 nm, corresponding respectively to π–π* transitions of aromatic rings, conjugated systems of phenolic acids, and cinnamoyl-type chromophores, consistent with the presence of both flavonoid and hydroxycinnamic structures. The ATR-FTIR spectrum further corroborated the prevalence of phenolic functionalities, showing intense stretching vibrations attributable to hydroxyl and carbonyl groups typical of polyphenolic matrices. In fact, the broad and intense bands at 3364 and 3271 cm^−1^ are attributed to O–H stretching vibrations of alcohols, phenols, and carboxylic acids, indicating the presence of intra- and intermolecular hydrogen bonding. The absorption peaks observed at 2934 cm^−1^ and 2851 cm^−1^ are attributed to the asymmetric and symmetric stretching vibrations of aliphatic C-H bonds present in CH_3_ and CH_2_ groups. The absorption at 1601 cm^−1^ is indicative of aromatic C=C stretching, while the band observed at 1510 cm^−1^ arises from a combination of aromatic C=C stretching and C–H bending, consistent with the presence of polyphenolic compounds such as flavonoids and phenolic acids. The signal at 1464 cm^−1^ can be attributed to C–H bending in aliphatic groups. Bands appearing at 1267 cm^−1^ and 1221 cm^−1^ are associated with C-O-H and C-O-C stretching vibrations, characteristic of phenolic or ether functionalities. Finally, the absorptions at 1136 and 1026 cm^−1^ suggest C-O stretching from alcohols and carbohydrate residues, indicating the possible presence of residual polysaccharides or phenolic glycosides.

The extract was further analyzed by ultra-high-performance liquid chromatography coupled to high-resolution Orbitrap mass spectrometry (UHPLC-Orbitrap HR-MS) to elucidate its chemical composition. The resulting high-resolution chromatographic profile revealed a complex mixture of phenolic compounds differing in polarity and structural class. Some metabolites were identified through targeted analysis using authentic standards, while additional compounds were annotated in untargeted mode based on accurate mass determination, isotopic distribution, and comparison with previously reported data on *Corylus avellana*-derived matrices.

To achieve a comprehensive chemical characterization of the hazelnut shell extract, both targeted and untargeted HR-MS approaches were therefore employed. The targeted method focused on the identification and quantification of ubiquitous and well-characterized polyphenols for which reference standards are available, enabling accurate quantification and reliable comparison with literature data. Conversely, the untargeted profiling was designed to explore the broader spectrum of less common or matrix-specific phenolic constituents, including diarylheptanoids and complex flavonoid derivatives characteristic of hazelnut by-products.

In the targeted analysis, performed in negative ionization mode ([M-H]^−^), a suite of major polyphenolic compounds was identified and quantified with high mass accuracy (|error| < 3.3 ppm). The chromatographic profile (Table 2) revealed the occurrence of several phenolic acids, quinic, protocatechuic, *p*-coumaric, chlorogenic, and ellagic acids, and flavonoids, including catechin, vitexin, luteolin 7-*O*-glucoside, quercetin and kaempferol glycosides, and apigenin 7-*O*-glucoside. These metabolites represent typical low-molecular-weight polyphenols contributing to the antioxidant potential of the extract. Quantitative results indicated that apigenin 7-*O*-glucoside was the most abundant compound (5.02 ppm), followed by quercetin 3-*O*-glucoside (1.55 ppm) and kaempferol 3-*O*-glucoside (1.33 ppm). The low limits of quantification (LOQ ≤ 0.02 ppm) and minimal mass errors confirm the analytical robustness and precision of the targeted HR-MS workflow.

Following the targeted evaluation of common polyphenols, an untargeted high-resolution mass spectrometry (HR-MS) analysis was performed to explore additional bioactive constituents in hazelnut shell extracts. Based on previous work [11], the analysis focused on a limited set of compounds, including two isomers of kaempferol 3-*O*-(*p*-coumaroyl)deoxyhexoside, previously reported in different parts of Mortarella and Camponica hazelnut varieties, as well as in the shells of the ‘Tonda di Giffoni’ cultivar. Additionally, the dihydrochalcone phloretin hexoside was detected, confirming the presence of this compound in hazelnut shells. Minor diarylheptanoids, such as giffonin U, giffonin V, and carpinontriol B, were also putatively identified (Table 3), consistent with their previous characterization in other hazelnut shells [11]. All compounds were detected in negative ionization mode ([M-H]^−^), with retention times and accurate masses in agreement with literature values. This targeted subset of untargeted metabolites highlights a chemically diverse profile of flavonoid glycosides and diarylheptanoids, providing both antioxidant potential and functional relevance for the development of bioactive polymer films. By focusing on these well-characterized metabolites, the untargeted HR-MS analysis complements the targeted profiling and supports the selection of polyphenolic fractions for bioactive applications.

### 3.2. Extraction and Functionalization of Hazelnut Shell Cellulose

**Figure 3:**
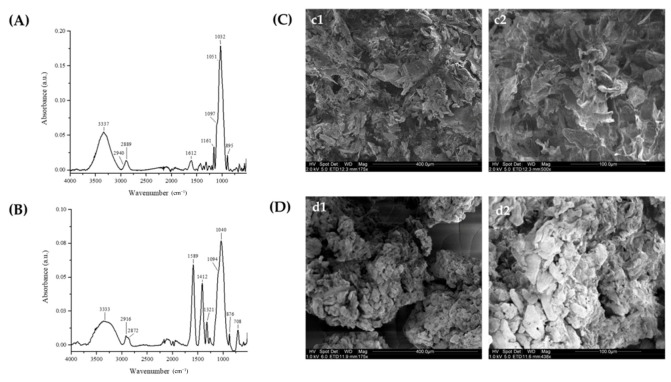
ATR-FTIR spectra of (**A**) hazelnut shell-derived cellulose, and (**B**) the resulting CMC. SEM micrographs of (**C**) cellulose and (**D**) CMC at two different magnifications: 400 μm (**c1**,**d1**) and 100 μm (**c2**,**d2**) scale bars.

The remaining solid residue was then processed for cellulose isolation. The delignification of lignocellulosic biomass using alkaline (NaOH) treatment is widely reported as it effectively disrupts ester and ether linkages within the lignin-carbohydrate complex structure [19]. In this study, the cellulose extraction was performed using reduced chemical utilization and reaction temperature as compared to conventional methods to maintain a more environmentally benign process. The alkaline treatment successfully loosened the rigid matrix of hazelnut shells, facilitating the release of cellulose embedded within the complex lignin-hemicellulose framework. However, a faint brownish tint was observed after alkali-treatment due to the persistence of trace lignin and residual pigments. To achieve high-purity cellulose, biomass was further treated with acidified sodium chlorite for bleaching. This oxidative step effectively eliminated residual chromophoric compounds and phenolic impurities, yielding bright white cellulose. Compared to hydrogen peroxide, sodium chlorite has demonstrated superior performance in removing chlorophyll derivatives and natural pigments, leading to high crystallinity and improved color quality [20,21]. The combination of mild alkaline delignification and NaClO_2_-bleaching thus resulted in high-purity cellulose with enhanced fibrillation and surface accessibility, as evidenced by SEM micrographs, further discussed below, supporting structural and thermal observations (Figure 3). The yield of hazelnut shell-derived cellulose was 22.7% (*w*/*w*) relative to the dry weight of hazelnut shell feedstock, reflecting the potential of the two-step extraction protocol. The cellulose yield obtained in this work (22.7% *w*/*w*, relative to the dry residual biomass after polyphenol extraction) is consistent with the cellulose content reported for hazelnut shells. Literature data indicate cellulose contents of approximately 26–34.6% in hazelnut shells, depending on cultivar, origin, and analytical method [22]. Lopes et al. [23] reported 28.9% cellulose in hazelnut shells and showed that oxidative pulping could produce high pulp yields; however, the resulting pulp was mainly composed of cellulose and xylans rather than pure cellulose. Therefore, the yield obtained here is acceptable, particularly since cellulose was recovered from biomass already subjected to polyphenol extraction in the proposed integrated biorefinery approach.

The obtained cellulose was subsequently functionalized to CMC in alkaline isopropanol medium using etherification via monochloroacetic acid. The structural and morphological transition confirmed a successful modification, showing the transformation of a dense, compact, fibrillar network of cellulose into an irregular, porous, and aggregated structure. These structural changes arise from the substitution of OH groups of cellulose with carboxymethyl moieties, disrupting the crystalline structure of cellulose [4,24]. This transformation resulted in a CMC yield of 0.91 g/g of cellulose, with a DS value of 0.77. The DS reflects the extent of carboxymethylation reaction along the cellulose backbone and is a key parameter influencing the viscosity, solubility, and overall functional properties of CMC [25]. The DS value less than 0.4 indicates that CMC swells but does not completely dissolve, while DS > 0.4 suggests that CMC is fully water-soluble [26]. The DS = 0.77 value of hazelnut shell-derived CMC falls within the typical range of biomass-derived CMC (0.4–0.9) [4,5,15], indicating a fully water-soluble polymer. A higher DS is linked to decreased crystallinity and enhanced chain mobility, resulting in biopolymeric films that are more uniform and transparent [1,4,5]. The obtained DS, with its structural and morphological features, together confirms the successful formation of CMC and highlights its potential as a sustainable material. The structural modifications occurring during carboxymethylation of the hazelnut shell cellulose were investigated by ATR-FTIR. Figure 3 shows the spectra recorded for the starting cellulose (panel A) and for the converted material (panel B), highlighting the main analogies and differences in the functional group region and fingerprint region that confirm the successful derivatization of the polysaccharide. Indeed, in both spectra, a broad and intense absorption band centered at approximately 3335 cm^−1^ was observed, corresponding to the stretching vibrations of OH groups engaged in intra- and intermolecular H-bonding within the polysaccharide network, as well as C-H asymmetric and symmetric stretching vibrations of aliphatic C-H bonds from the glucose rings around 2900–2800 cm^−1^. Following the carboxymethylation reaction, new bands were observed at 1589 and 1412 cm^−1^, attributable to the asymmetric and symmetric stretching vibrations of the carboxylate anion, whose appearance and relative intensity provide clear evidence of successful carboxymethylation [4].

Other characteristic signals of the polysaccharide structure are evident in the fingerprint region. The strong absorption centered at 1032 cm^−1^ is related to the C-O stretching vibrations of the C-O-C glycosidic linkage, while the bands at 1161 and 1097 cm^−1^ are ascribable to asymmetric and symmetric stretching of the ether linkages within the β-1,4-glycosidic structure. They were also detected in the case of CMC, although slightly shifted at higher wavenumber values, likely due to the cellulose functionalization. The β-glycosidic linkages among polysaccharide monomers were highlighted by the peak at 895 cm^−1^ in the cellulose sample, whose vibration frequency decreased to 876 cm^−1^ in the CMC sample, further confirming some structural rearrangements after etherification [4,5].

**Figure 4:**
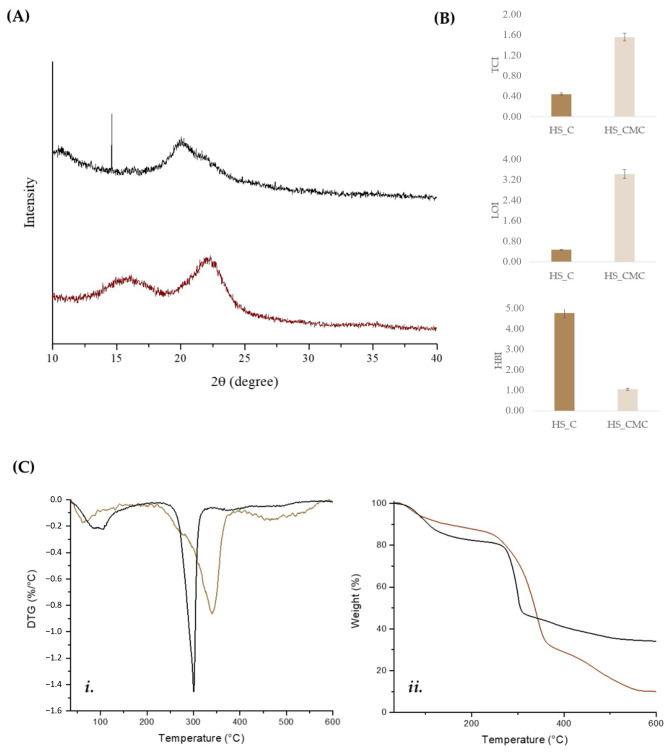
(**A**) X-ray diffraction (XRD) patterns of HS-C (brown) and HS-CMC (black). (**B**) Bar graphs showing the average values (mean ± SD) of crystallinity-related indices: Total Crystallinity Index (TCI), Lateral Order Index (LOI), and Hydrogen Bond Intensity (HBI), calculated from ATR-FTIR spectra. Data represents the mean of three independent measurements. (**C**) Representative *i*. thermogravimetric (TGA) and *ii*. derivative thermogravimetric (DTG) curves of HS-C (brown) and HS-CMC (black) samples. HS-C: T_initial_ 143 °C; T_max_ 340 °C; T_50%_ 337 °C; T_final_ 381 °C; residual mass 10%. HS-CMC: T_initial_ 190 °C; T_max_ 300 °C; T_50%_ 305 °C; T_final_ 347 °C; residual mass 34%.

HS-C, as evidenced by X-ray diffraction, exhibited the typical pattern of cellulose I (Figure 4, panel A). The diffractogram displayed two main reflections at 15.9° and 22.2°, confirming the presence of a partially ordered microstructure. The crystallinity index, calculated using the Segal method from the intensity of the 22.2° reflection and the minimum between the main reflections, was 40.9%, indicating a moderate degree of structural order. This result is consistent with the ATR-FTIR-derived indices calculated for HS-C, which showed relatively low TCI (0.44) and LOI (0.47) values, suggesting limited lateral order and the presence of a substantial amorphous fraction (Figure 4, panel B). After carboxymethylation, HS-CMC showed a different trend in the ATR-FTIR-derived crystallinity-related indices (Figure 4, panel B). Indeed, TCI increased from 0.44 to 1.55 and LOI from 0.47 to 3.43, while HBI decreased from 4.82 to 1.06. These variations should not be interpreted as a direct increase in the absolute crystallinity of CMC, but rather as changes in the relative intensity of characteristic ATR-FTIR bands associated with chain packing, hydrogen bonding, and structural rearrangement after etherification. In particular, the decrease in HBI indicates a modification and weakening of the original hydrogen-bonding network, consistent with the introduction of carboxymethyl groups and the disruption of the native cellulose structure.

The thermal behavior and degradation profiles of HS-C and HS-CMC were investigated by thermogravimetric analysis (TGA) and derivative thermogravimetry (DTG). The black curve, corresponding to HS-CMC (Figure 4, panel C), showed differences in the thermal resistance compared to the brown curve representing the untreated sample (HS-C). Both materials showed a major weight-loss event around 300 °C, associated with the decomposition of the polysaccharide backbone, and this transition appeared as a sharp DTG peak, more intense and slightly shifted in HS-CMC. Beyond the main degradation step, the quantitative parameters extracted from TGA provide a clearer picture of the effect of chemical modification. HS-CMC displayed a higher onset temperature (T_initial_ = 190 °C) than HS-C (143 °C), suggesting increased resistance to the initial stages of thermal degradation. Conversely, the temperature at 50% mass loss (T_50%_) decreased from 337 °C in HS-C to 305 °C in HS-CMC, indicating that once the main degradation process begins, the modified structure undergoes mass loss at a lower temperature. A similar trend was observed for T_final_, which decreased from 381 °C to 347 °C, reflecting a lower energy requirement for complete degradation of the modified matrix. The most striking difference was found in the residual mass at 600 °C, which increased from 10% in HS-C to 34% in HS-CMC, indicating the formation of a more thermally stable char. This behavior suggests that the introduction of carboxymethyl groups promotes structural rearrangements that enhance char formation and thermal robustness.

To further support these structural and thermal observations, surface morphologies of hazelnut shell cellulose and resulting CMC were examined using scanning electron microscopy (SEM) at different magnifications (Figure 3). Panel C (c1 and c2 micrographs) presents hazelnut shell-derived cellulose, whereas panel D (d1 and d2 images) corresponds to the resulting CMC. The SEM micrographs of cellulose reveal a compact and fibrillar morphology, with layered sheet-like fragments that are tightly packed and closely interconnected. The overall aspect is typical of purified cellulose microfibrils, where the hydrogen-bonding network confers structural rigidity and dense packing. The relatively homogeneous texture suggests that non-cellulosic components such as lignin and hemicellulose were effectively removed during the extraction steps, yielding a clean polysaccharide surface. The layered structure observed at higher magnification (image c2, Figure 3C) further confirms the presence of well-organized fibrils with limited porosity. These features align with the previous studies on cellulose derived from nut shells and other lignocellulosic biomasses [4,5,27], confirming the successful extraction of relatively pure cellulose fibers. In contrast, the morphology of synthesized CMC (Figure 3D) shows a clear transformation. The fibrous hazelnut shell cellulose is replaced by more aggregated and compact granules with a rough and porous surface. At lower magnification (micrograph d1), the CMC particles appear as irregular clusters, while at higher magnification (micrograph d2), the surface presents clear signs of swelling and partial disintegration of the fibrous framework, which is attributed to the occurrence of the etherification reaction. The substitution of OH disrupts inter- and intramolecular H-bonding, resulting in the formation of a less ordered and more amorphous structure [24,28]. The disruption/reorganization of the native cellulose structure after carboxymethylation is in line with improved swelling capacity and water affinity, consistent with its enhanced carboxymethylation and hydrophilicity, which also confirms the successful chemical modification of cellulose. These results suggest the potential suitability of hazelnut shell-derived CMC for different bio-sector applications where high surface area and water interaction are critical, including hydrogel manufacturing, adsorbents, and biodegradable packaging films.

### 3.3. Characterization of Newly Produced Bioplastic Films

#### 3.3.1. Structural Characterization

**Figure 5:**
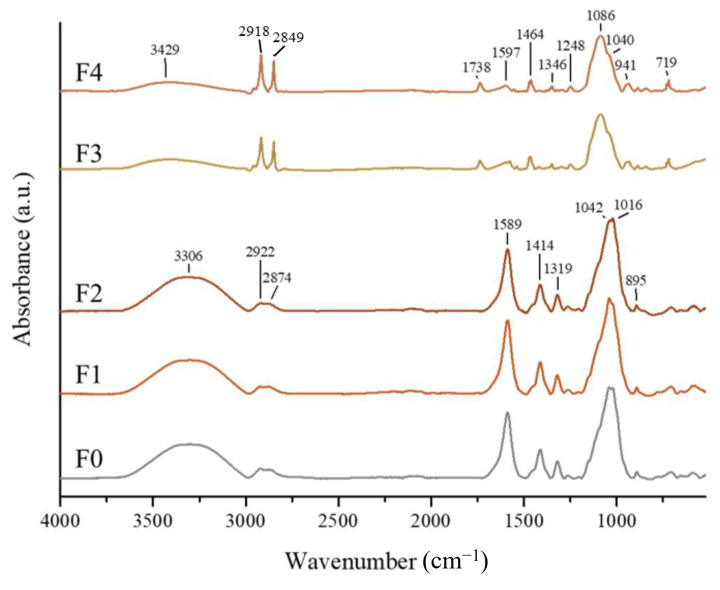
ATR-FTIR spectra of hazelnut shell polyphenol-based films. F0: control (without polyphenolic extract); F1: 40 mg/100 mL polyphenolic extract; F2: 80 mg/100 mL polyphenolic extract; F3: 40 mg/100 mL polyphenolic extract + 400 mg/100 mL carnauba wax (CW); F4: 80 mg/100 mL polyphenolic extract + 400 mg/100 mL CW.

Figure 5 shows the ATR-FTIR spectra of bioplastic films F0–F4 prepared with hazelnut shell carboxymethyl cellulose (HS-CMC) as the main polymer matrix, plasticized with sorbitol, and variably enriched with hazelnut shell polyphenolic fractions (HS-PFs) and with/without the addition of the carnauba wax emulsion. The spectral profiles revealed the characteristic functional groups of the polymeric matrix, as previously discussed. In fact, the spectrum of the control film (F0), containing only CMC and sorbitol, displayed the typical absorptions of carboxymethyl cellulose. Upon incorporation of the (poly)phenolic extract at different concentrations (F1 and F2), the general spectral profile remained similar, indicating that the polymeric structure was preserved. The relative intensity of the carboxylate bands at 1589 cm^−1^ and 1414 cm^−1^ slightly increased, in accordance with partial ion–dipole interactions between the phenolic components and the CMC carboxylate moieties. These changes confirmed a certain degree of compatibility and molecular interaction between HS-PFs and the CMC matrix. On the contrary, the films containing the carnauba wax emulsion (F3 and F4) exhibited new and distinctive features, highlighting the formation of a more complex, amphiphilic film network. Indeed, hydrogen bonding, predominating between CMC, sorbitol, and HS-PFs, was markedly hindered by hydrophobic interactions that dominated when wax emulsion was present. Moreover, a marked band appeared at 1738 cm^−1^, attributed to the C=O stretching vibration of ester carbonyl groups in the long-chain wax esters. Additionally, the absorption peaks at 2918 and 2849 cm^−1^ became sharper and more intense, reflecting the contribution of aliphatic C-H stretching from the wax hydrocarbon chains [4]. The weak band at 3429 cm^−1^ corresponded to residual hydroxyl stretching from minor alcohol or acid groups in the wax and polymer components. The bands observed at 1597–1346 cm^−1^ further suggested overlapping contributions from phenolic aromatic C=C stretching and C-O vibrations of esters and ethers in the composite matrix. The region between 1248 and 1040 cm^−1^ retained the C-O-C stretching signatures of the cellulose skeleton, while the slight decrease in their intensity in F3 and F4 indicated partial physical coating of the polysaccharide surface by carnauba wax emulsion, leading to reduced exposure of hydrophilic groups. The appearance of a small band at 719 cm^−1^ in these formulations likely corresponded to long-chain methylene rocking vibrations, another clear indicator of wax incorporation [29].

#### 3.3.2. Morphological Characterization—SEM

**Figure 6:**
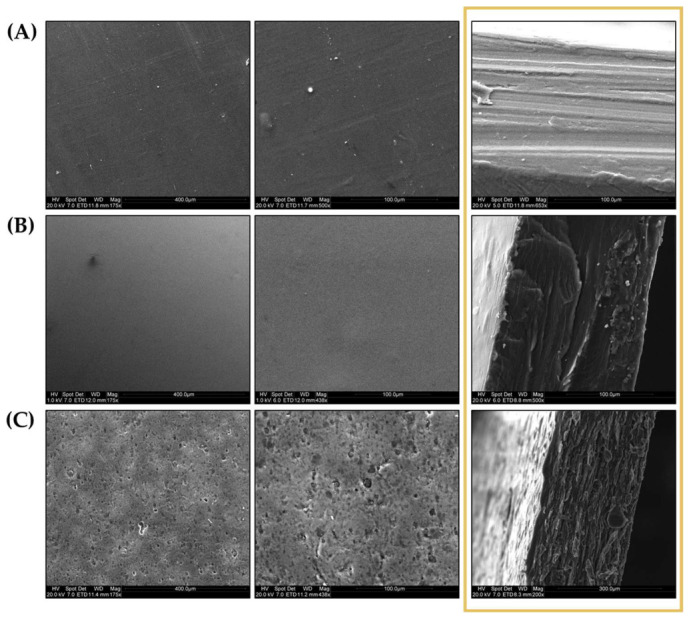
Surface representative morphologies of F0 (**A**), F2 (**B**), and F4 (**C**), obtained by SEM. Cross-sectional images are depicted in the yellow box.

The surface and cross-sectional morphologies of bioplastic films obtained from hazelnut shell-derived CMC were examined to investigate the effect of additives on film homogeneity and microstructural changes. The SEM images of control film F0 (without additives), active film F2 (80 mg/100 mL HS-PFs), and composite film F4 (80 mg/100 mL HS-PFs + 400 mg/100 mL carnauba wax) are shown in Figure 6. SEM images of the control film revealed smooth and uniform surface morphology with minimal defects or pores. The surface homogeneity suggested excellent film-forming capabilities of the pure CMC, resulting in dense and compact films. The absence of granular domains or voids demonstrated that sorbitol was effectively incorporated as a plasticizer, enhancing surface regularity [30].

The cross-sectional image further confirmed this feature, showing well-packed layers without visible cracks or phase separation, indicating that the film formation process produced a cohesive and flexible matrix. The incorporation of HS-PFs into the CMC matrix induced slight changes in film morphology. The surface still appears relatively smooth, but a few irregularities and fine granulations can be observed at higher magnification, likely resulting from partial aggregation of phenolic compounds or their localized interactions with the CMC matrix. The internal structure remains continuous, but slightly more heterogeneous, suggesting that the phenolic molecules were partially embedded within the polymeric network through hydrogen bonding. This moderate roughening can also indicate enhanced intermolecular interactions (CMC-PFs and PFs-PFs), which may influence mechanical and barrier properties by reinforcing the film structure while reducing its transparency.

The incorporation of carnauba wax emulsion (F4) further changed the morphology, as previously observed [4]. The surface of F4 appears uneven and granular with visible discontinuities and aggregates, indicative of partial phase separation between the hydrophobic wax and the hydrophilic polymer matrix. At higher magnification, the micrographs show distinct waxy structures dispersed within or coating the surface, forming discontinuous domains typical of lipid incorporation in biopolymer films. Cross-sectional images further confirmed a heterogeneous morphology with phase boundaries and stratified regions, suggesting that wax droplets partially coalesced during drying. Although this morphology does not indicate complete CMC–wax compatibility, the presence of wax-rich domains and lamellar/patch-like regions may contribute to the reduced water sensitivity of the film by introducing hydrophobic barriers and increasing the tortuosity of water diffusion pathways.

This morphological rearrangement correlates with the ATR-FTIR results, which confirmed the appearance of ester C=O at 1738 cm^−1^ and aliphatic CH_2_ stretching bands associated with wax incorporation.

**Figure 7:**
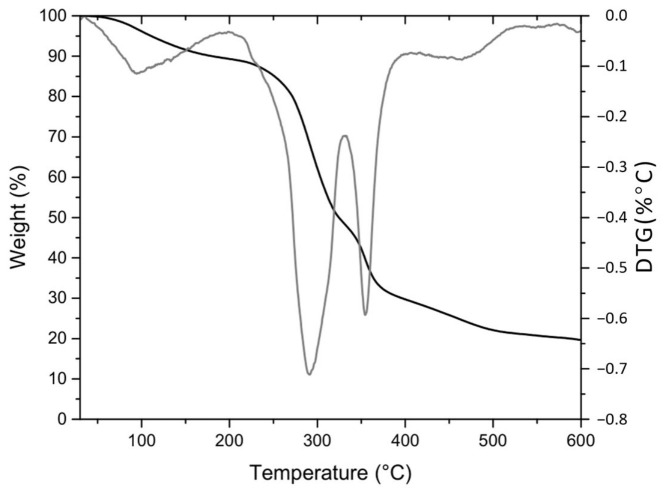
Representative thermogravimetric and derivative thermogravimetric curves of F4.

Building on these morphological observations, the thermal behavior of F4 film was examined through TGA and DSC analyses to clarify how such structural rearrangements influence film stability (Figure 7). Briefly, the TGA curve showed an initial mass loss at approximately 202 °C (T_initial_), associated with the onset of thermal degradation. The main decomposition event occurred at 356 °C (T_max_), corresponding to the breakdown of the polysaccharide network and the thermolabile components of the extract. The T_50%_ was 324 °C, while complete degradation was reached at 399 °C (T_final_). The film left a residual mass of 20% at 600 °C, indicating the formation of a moderately stable char, likely promoted by the presence of polyphenolic compounds and wax emulsion. The DTG thermogram supported these findings, revealing an endothermic transition at lower temperatures associated with moisture loss and partial melting of wax domains, followed by broader thermal events reflecting structural rearrangements and progressive degradation of the composite. Overall, the combined TGA/DTG results indicate that the incorporation of carnauba wax emulsion and polyphenolic extract modifies both the onset and progression of thermal transitions, suggesting interactions within the matrix that influence the film’s thermal stability.

#### 3.3.3. Physical Properties of Films

Different physical properties of bioplastic films, including thickness, color analysis, transparency index, moisture content, solubility, water vapor transmission rate (*WVTR*), and water vapor permeability (*WVP*), were determined. Visual appearance and chroma parameters (L*, a*, b*, C*, and h) are reported in Table 4, while other data regarding physical properties are summarized in Table 5.

The control film (F0) showed good homogeneity with an 8.51 ± 0.00 transparency index, 65.91 ± 2.73 whiteness index, and 47.35 ± 5.23 yellowness index. The film (F0) showed a highly hydrophilic nature with a 6.60 ± 0.73% moisture content and 94.38 ± 0.31% aqueous solubility. Further, the *WVTR* values were 18.05 ± 0.04 g/m^2^×h and 24.42 ± 0.02 g/m^2^×h after 24 h and 48 h, respectively. The addition of polyphenolic extract resulted in a slightly reduced transparency [*TI*: 7.98 ± 0.01 (F1) and 7.34 ± 0.01 (F2)], with decreasing whiteness index [46.53 ± 5.77 (F1) and 36.31 ± 0.62 (F2)] and increasing yellowness [87.64 ± 14.89 (F1) and 113.42 ± 2.42 (F2)] index. The addition of polyphenolic compounds significantly reduced film solubility [from 94.38 ± 0.31% (F0) to 59.74 ± 1.44% (F1), and 30.92 ± 1.03 (F2)], which could be attributed to the good dispersibility of polyphenolic compounds leading to a more compact and denser structure. However, to further improve water resistance, the incorporation of carnauba wax emulsion was carried out, which compromised transparency [*TI*: 1.83 ± 0.01 (F3) and 2.01 ± 0.01 (F4)], which can be related to light scattering caused by dispersed wax-rich domains and the more heterogeneous morphology observed by SEM. At the same time, the film solubility was significantly reduced to 19.12 ± 0.61% (F3) and 12.74 ± 1.14 (F4). This behavior should be interpreted as the result of multiple concurrent factors, including the hydrophobic nature of carnauba wax, the presence of wax-rich domains, partial phase separation, film thickness, and the possible increase in tortuosity of water diffusion pathways within the HS-CMC matrix. Therefore, the improved water-resistance-related properties of the wax-containing films could be attributed to the overall microstructured wax-emulsion formulation rather than to wax hydrophobicity alone.

#### 3.3.4. Mechanical Properties of Films

The mechanical properties are critical to assess material suitability for food packaging during transportation and handling [1]. The mechanical parameters (tensile strength, Young’s modulus, and percent elongation at break) are summarized in Table 6. The control film (F0) exhibited a tensile strength of 49.98 ± 0.03 MPa, Young’s modulus of 36.95 ± 1.11 MPa, and elongation at break of 16.23 ± 0.63%. The incorporation of polyphenolic compounds at 40 mg/100 mL (F1) and 80 mg/100 mL (F2) significantly enhanced the tensile strength as compared to the control (F0). Film F1 showed a tensile strength of 58.47 ± 0.30 MPa, Young’s modulus of 33.69 ± 2.16 MPa, and elongation at break of 20.87 ± 1.23%, while F2 recorded a maximum tensile strength of 77.61 ± 2.66 MPa, Young’s modulus of 43.75 ± 0.18 MPa, and elongation at break of 21.29 ± 0.64%. These results clearly suggest that the addition of polyphenolic compounds to hazelnut shell-derived CMC matrix reinforces the films, likely due to the formation of secondary interactions such as hydrogen bonding between the OH group of CMC and polyphenolic extract. Such interactions have been previously reported to improve mechanical integrity and stress transfer within polysaccharide matrices [16,31]. Interestingly, the polyphenolic extract addition showed a dual effect on Young’s modulus by first slightly decreasing in F1 (33.69 ± 2.16 MPa) and then significantly increasing in F2 (43.75 ± 0.18 MPa). The decrease in Young’s modulus in F1 suggests that the extract at lower concentration may have acted as a plasticizer, increasing mobility in polymer chains. However, at higher extract loading (F2), the extract shows a transition from plasticizing to reinforcing agent. This concentration-dependent behavior is attributed to the formation of a cohesive network of polymer-polyphenols, providing greater resistance to deformation [32].

The incorporation of carnauba wax emulsion significantly altered the film stiffness and flexibility. Film F3 (40 mg/100 mL extract + 400 mg/100 mL carnauba wax) exhibited a tensile strength of 52.12 ± 2.70 MPa and a reduced Young’s modulus (23.09 ± 7.08 MPa) with the highest elongation at break (28.20 ± 7.25%) among all samples. This decreased stiffness and increased elongation indicate that the carnauba wax domains acted as a soft phase, disrupting intermolecular H-bonding and enhancing polymer chain mobility. However, at higher extract loading, the incorporation of carnauba wax emulsion (F4) resulted in significantly improved tensile strength (75.09 ± 5.61 MPa) and Young’s modulus (40.27 ± 1.38 MPa) with moderate elongation (22.41 ± 2.44%). At higher extract loading, the wax-containing films showed features consistent with improved matrix-additive interactions. This behavior may be associated with the presence of some constituents in the extract, which could facilitate interactions between the hydrophilic CMC network and the dispersed wax phase. Overall, the mechanical behavior demonstrates a clear structure–property relationship directed by the relative extract loading. The extract alone reinforces the CMC matrix, enhancing tensile strength and elongation, while carnauba wax emulsion introduces flexibility but reduces stiffness. Among all, F2 showed superior load-bearing capacity, suggesting possible applications in food packaging. However, F3 exhibited the highest elongation and soft texture, which may be desirable in flexible food wrappings. The film F4 achieved a balance between F2 and F3, combining higher strength with moderate flexibility, which is specifically desirable for biodegradable packaging systems with superior functional performance, such as UV-blocking and antioxidative potential, as described in the next section.

#### 3.3.5. Bio-Functional Performance of Films

**Figure 8:**
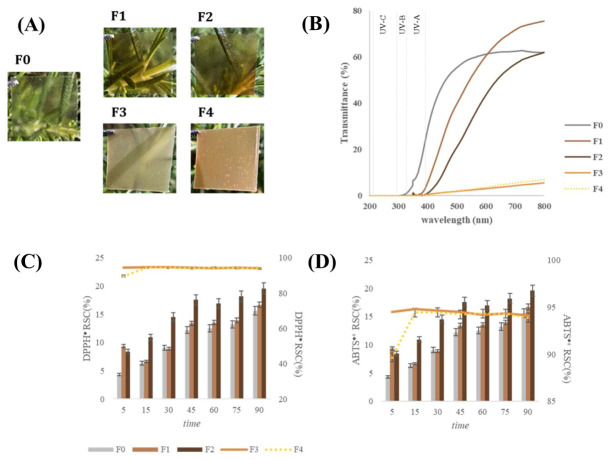
(**A**) Physical appearance of films, (**B**) UV-Vis spectra (200–800 nm) indicating UV-absorbing/blocking potential of films, (**C**) DPPH- and (**D**) ABTS radical-scavenging activities of films produced from hazelnut shell-derived CMC with/without hazelnut shell extract.

To assess the bio-functional performance of the developed films, UV-blocking and antiradical (DPPH and ABTS) assays were performed (Figure 8). The control film (F0) exhibited the highest transmittance across the UV region, confirming its poor UV-shielding ability. In contrast, the incorporation of polyphenolic extract significantly reduced UV transmittance, particularly between 200–300 nm, with a concentration-dependent effect. Films F1 and F2 showed progressively enhanced UV-blocking performance, attributed to the strong absorption capacity of phenolic compounds. The addition of carnauba wax emulsion in formulations F3 and F4 further decreased UV transmittance, leading to the most effective protection against UV radiation. However, this improvement in UV-blocking capacity was accompanied by a noticeable reduction in transparency, as evident from the optical appearance of films (Figure 8, panels A and B). Overall, the integration of bioactive and hydrophobic components effectively enhanced UV-shielding behavior, making the films suitable for protecting light-sensitive and perishable food products.

The antioxidant activity, evaluated through DPPH and ABTS radical scavenging assays, revealed that films containing both polyphenolic extract and carnauba wax emulsion (F3 and F4) exhibited the strongest antiradical performance (Figure 8, panels C and D). Against DPPH, F3 and F4 achieved approximately 40% scavenging after 5 min and reached up to 80% after 90 min, while their activity toward the ABTS radical cation was even more pronounced, showing nearly complete radical reduction within the first 5 min. Interestingly, the presence of carnauba wax emulsion did not diminish but rather enhanced the antioxidant capacity of the films. This effect can be explained by the intrinsic bioactive composition of carnauba wax, which contains phenol and flavonoid-like constituents with recognized antioxidant properties [33]. The wax therefore contributes not only as a hydrophobic and structural additive but also as a secondary source of antioxidant compounds. Moreover, the hydrophobic matrix of carnauba wax likely protects polyphenolic molecules from oxidative degradation and light-induced decomposition, thus preserving their long-term efficacy. A possible additional contribution from wax-related components cannot be excluded, although specific control formulations would be required to confirm this effect.

Taken together, these results highlight that the co-presence of hazelnut shell polyphenols and carnauba wax emulsion generates a bioactive system that combines effective UV-blocking behavior with strong and sustained antioxidant activity. Such multifunctional performance highlights the potential of these films for active food-packaging applications, warranting further investigation in real food systems.

## 4. Conclusions

This study demonstrates a fully integrated and sustainable valorization pathway for hazelnut shell waste, enabling the sequential recovery of bioactive polyphenols and cellulose, two high-value fractions typically discarded during processing. The extracted cellulose was efficiently converted into carboxymethyl cellulose (CMC) under mild and environmentally friendly conditions, yielding a renewable polymeric matrix suitable for the fabrication of functional films. Incorporation of hazelnut shell-derived polyphenolic extracts modified the film mechanical strength, UV-blocking capacity, and antioxidant performance in a concentration-dependent manner, driven by strong hydrogen-bonding and secondary interactions between CMC and phenolic constituents. The high purity and crystallinity of the hazelnut-derived cellulose were consistent with the observed compatibility with the bioactive fraction, supporting the development of structurally robust and functionally enriched materials. Additional reinforcement with carnauba wax emulsion further improved water resistance and modulated film flexibility, while its intrinsic antioxidant components likely contributed to amplify overall bioactivity. Collectively, these findings highlight the dual potential of hazelnut shells as a source of both structural (cellulose) and functional (polyphenolic) compounds, demonstrating a circular biorefinery approach capable of transforming agricultural residues into bioactive packaging materials aligned with the principles of green chemistry and sustainable design. From a process perspective, the proposed biorefinery approach enabled the recovery of both functional and structural fractions from hazelnut shell biomass. Based on the experimentally determined recoveries reported throughout the manuscript, the integrated process yielded approximately 0.44 g of the purified polyphenolic fraction and 22.7 g of cellulose-rich material per 100 g of hazelnut shells, corresponding to about 20.7 g of HS-CMC after carboxymethylation. Starting from this awareness, further studies are required to assess migration behavior, safety and regulatory compliance, biodegradability, storage stability, antimicrobial efficacy, and performance in contact with real food systems. In addition, future work should evaluate the economic feasibility of the process, including reagent and solvent recovery, chemical consumption, scalability, and overall production costs.

## Figures and Tables

**Table 1:** Film composition and designated IDs of hazelnut shell CMC-based films with/without carnauba wax and bioactive extract to investigate their effect on different physicochemical and functional properties.

Film IDs	HS-CMC (g/100 mL)	Sorbitol (g/100 mL)	HS-PF (mg/100 mL)	Carnauba Wax * (mg/100 mL)
F0	2.80	1.20	–	–
F1	2.80	1.20	40	–
F2	2.80	1.20	80	–
F3	2.80	1.20	40	400
F4	2.80	1.20	80	400

* added as an emulsion with agar (200 mg/100 mL) and Tween 40 (400 µL/100 mL).

**Table 2:** Targeted HR-MS analysis (negative ion mode) of polyphenolic compounds from hazelnut shell extract. LOQ = Limit of Quantification, the lowest compound concentration that can be quantitatively detected with acceptable precision and accuracy.

Compounds	LOQ (ppm)	RT (min)	[M-H]^−^ (*m*/*z*)	Found Mass (*m*/*z*)	Error (ppm)	Chemical Formula	Average Polyphenol Amount (ppm)
Quinic acid	0.0049	0.71	191.0551	191.0553	−1.047	C_7_H_12_O_6_	0.085
Protocatechuic acid	0.0195	3.68	153.0181	153.0186	−3.268	C_7_H_6_O_4_	0.073
Catechin	0.0049	3.98	289.0722	289.0721	0.346	C_15_H_14_O_6_	0.525
Chlorogenic acid	0.0049	4.04	353.0885	353.0878	1.983	C_16_H_18_O_9_	0.093
Vitexin	0.0024	4.15	431.0982	431.0983	−0.232	C_21_H_20_O_10_	0.268
Luteolin 7-*O*-glucoside	0.0049	4.26	447.0942	447.0936	1.342	C_21_H_20_O_11_	0.253
*p*-coumaric acid	0.0195	4.28	163.0399	163.0394	3.067	C_9_H_8_O_3_	0.295
Rutin	0.0195	4.40	609.1465	609.1467	−0.328	C_27_H_32_O_17_	0.108
Quercetin 3-*O*-glucoside	0.0049	4.41	463.0886	463.0888	−0.432	C_21_H_20_O_12_	1.553
Kaempferol 3-*O*-glucoside	0.0049	4.52	447.0942	447.0936	1.342	C_21_H_20_O_11_	1.333
Ellagic acid	0.0049	4.54	300.9991	300.9992	−0.332	C_14_H_6_O_8_	0.400
Apigenin-7-*O*-glucoside	0.0049	4.65	431.0991	431.0986	1.160	C_21_H_20_O_10_	5.018
Quercetin	0.0049	4.77	301.036	301.0351	2.990	C_15_H_10_O_7_	0.055

**Table 3:** Untargeted HR-MS analysis of selected polyphenolic compounds in hazelnut shell extracts. Compounds were putatively identified in negative ionization mode ([M-H]^−^) based on accurate mass, retention time, and comparison with literature data [11]. The table reports the compound name, retention time (RT, min), theoretical and measured deprotonated masses ([M-H]^−^, *m*/*z*), mass error (ppm), and chemical formula.

Compounds	RT (min)	[M-H]^−^ (*m*/*z*)	Found Mass (*m*/*z*)	Error (ppm)	Chemical Formula
Giffonin U	8.99	359.1136	359.1138	0.557	C_19_H_20_O_7_
Carpinontriol B	10.45	343.1187	343.1188	0.2914	C_19_H_18_O_9_
phloretin hexoside	10.97	435.1297	435.1296	−0.230	C_21_H_24_O_10_
Giffonin V	11.99	327.1238	327.1237	−0.3057	C_19_H_20_O_5_
Kaempferol 3-*O*-(*p*-coumaroyl)deoxyhexoside I	14.63	577.1351	577.1354	0.5198	C_30_H_24_O_10_
Kaempferol 3-*O*-(*p*-coumaroyl)deoxyhexoside II	14.78	577.1351	577.1357	1.040	C_30_H_24_O_10_

**Table 4:** Chroma parameters (L*, a*, b*, C*, and *h*) of HS-CMC-based films with/without polyphenolic extract and carnauba wax emulsion. Different superscript letters (a, b, c, d, and e) within a column denote statistically significant differences (*p* < 0.05), whereas identical letters indicate no significant difference (*p* > 0.05) between treatment means.

Film IDs	Chroma Parameters				
	L*	a*	b*	C*	*h*
**F0**	77.63 ± 1.45 ^a^	−0.92 ± 0.13 ^e^	25.7 ± 2.36 ^c^	25.72 ± 2.36 ^c^	92.07 ± 0.48 ^a^
**F1**	65.13 ± 1.88 ^b^	7.12 ± 2.56 ^c^	39.86 ± 5.64 ^b^	40.50 ± 6.00 ^b^	80.03 ± 2.18 ^b^
**F2**	58.12 ± 0.08 ^c^	13.16 ± 0.40 ^a^	46.16 ± 1.05 ^a^	47.99 ± 0.90 ^a^	74.08 ± 0.81 ^c^
**F3**	67.93 ± 0.18 ^b^	4.52 ± 0.04 ^d^	24.79 ± 0.37 ^c^	25.20 ± 0.37 ^c^	79.67 ± 0.06 ^b^
**F4**	57.84 ± 0.08 ^c^	9.68 ± 0.33 ^b^	30.43 ± 0.48 ^c^	31.94 ± 0.36 ^c^	72.37 ± 0.83 ^d^

**Table 5:** Physical characteristics of HS-CMC-based films with/without polyphenolic extract and carnauba wax. Results are represented as mean ± standard deviation (*n* = 3). Different superscript letters (a, b, c, d, and e) within a column denote statistically significant differences (*p* < 0.05), whereas identical letters indicate no significant difference (*p* > 0.05) between treatment means.

Film IDs	Film Thickness (mm)	Visual Appearance			Moisture Content (%)	Solubility (%)	*WVTR* ^4^ (g/m^2^× h)		*WVP* ^5^ [g × mm/(m^2^ × h × kPa)]	
		*WI* ^1^	*YI* ^2^	*TI* ^3^			*WVTR*_24_	*WVTR*_48_	*WVP*_24_	*WVP*_48_
F0	0.21 ± 0.01 ^a^	65.91 ± 2.73 ^a^	47.35 ± 5.23 ^a^	8.51 ± 0.00 ^a^	6.60 ± 0.73 ^a^	94.38 ± 0.31 ^a^	18.05 ± 0.04 ^c^	24.42 ± 0.02 ^a^	2.39 ± 0.01 ^d^	3.23 ± 0.00 ^a^
F1	0.22 ± 0.01 ^a^	46.53 ± 5.77 ^b^	87.64 ± 14.89 ^a^	7.98 ± 0.01 ^b^	2.97 ± 1.04 ^b^	59.74 ± 1.44 ^b^	20.69 ± 2.18 ^bc^	16.81 ± 0.03 ^bc^	2.92 ± 0.31 ^cd^	2.37 ± 0.00 ^cd^
F2	0.22 ± 0.01 ^a^	36.31 ± 0.62 ^b^	113.42 ± 2.42 ^a^	7.34 ± 0.01 ^c^	6.52 ± 0.63 ^a^	30.92 ± 1.03 ^c^	36.16 ± 0.24 ^a^	21.75 ± 0.00 ^ab^	5.09 ± 0.03 ^a^	3.06 ± 0.00 ^ab^
F3	0.27 ± 0.01 ^b^	59.21 ± 0.37 ^a^	52.14 ± 0.91 ^a^	1.83 ± 0.01 ^d^	3.77 ± 0.74 ^b^	19.12 ± 0.61 ^d^	22.94 ± 0.07 ^b^	14.23 ± 0.03 ^c^	3.96 ± 0.01 ^b^	2.45 ± 0.01 ^bc^
F4	0.29 ± 0.01 ^b^	47.11 ± 0.15 ^b^	75.16 ± 1.08 ^a^	2.01 ± 0.01 ^d^	1.45 ± 0.10 ^c^	12.74 ± 1.14 ^e^	12.37 ± 0.00 ^d^	12.26 ± 0.04 ^d^	2.26 ± 0.00 ^d^	2.24 ± 0.01 ^d^

^1^ WI: whiteness index; ^2^ YI: yellowness index; ^3^ TI: transparency index; ^4^ WVTR: water vapor transmission rate; ^5^ WVP: water vapor permeability.

**Table 6:** Mechanical properties (tensile strength, Young’s modulus, and percent elongation at break) of hazelnut shell-derived CMC-based films with/without active reinforcements. Different superscript letters (a, b) within a column denote statistically significant differences (*p* < 0.05), whereas identical letters indicate no significant difference (*p* > 0.05) between treatment means.

Film IDs	Tensile Strength (MPa)	Young’s Modulus (MPa)	Elongation at Break (%)
F0	49.98 ± 0.03 ^a^	36.95 ± 1.11 ^a^	16.23 ± 0.63 ^a^
F1	58.47 ± 0.30 ^a^	33.69 ± 2.16 ^a^	20.87 ± 1.23 ^a^
F2	77.61 ± 2.66 ^b^	43.75 ± 0.18 ^a^	21.29 ± 0.64 ^a^
F3	52.12 ± 2.70 ^a^	23.09 ± 7.08 ^a^	28.20 ± 7.25 ^a^
F4	75.09 ± 5.61 ^b^	40.27 ± 1.38 ^a^	22.41 ± 2.44 ^a^

## Data Availability

The original contributions presented in this study are included in the article. Further inquiries can be directed to the corresponding author.
